# Diethylnitrosamine (DENA) recapitulates formation of hepatic angiosarcoma in pigs

**DOI:** 10.1371/journal.pone.0214756

**Published:** 2019-05-16

**Authors:** Sonja M. Kessler, Bettina Leber, Jessica Hoppstädter, Nicole Golob-Schwarzl, Eva M. Hofer, Christina S. Schultheiss, Hans-Jörg Mischinger, Bernadette Liegl-Atzwanger, Carolin Lackner, Philipp Stiegler, Johannes Haybaeck

**Affiliations:** 1 Diagnostic & Research Center for Molecular BioMedicine, Institute of Pathology, Medical University of Graz, Graz, Austria; 2 Department of Pharmacy, Pharmaceutical Biology, Saarland University, Saarbrücken, Germany; 3 Department of Surgery, Medical University of Graz, Graz, Austria; 4 Institute of Dermatology and Venerology, Medical University of Graz, Graz, Austria; 5 Department of Pathology, Medical Faculty, Otto von Guericke University Magdeburg, Magdeburg, Germany; 6 Department of Pathology, Neuropathology and Molecular Pathology, Medical University of Innsbruck, Innsbruck, Austria; University of Navarra School of Medicine and Center for Applied Medical Research (CIMA), SPAIN

## Abstract

**Background & aim:**

Primary hepatic angiosarcoma is a rare tumor with poor prognosis. The aim of this study was to generate a new angiosarcoma model to improve research on hepatic angiosarcoma.

**Methods:**

Pigs *sus scrofa* were treated with different regimens of diethylnitrosamine (DENA). Tissues were analyzed by histology and immunohistochemistry. Serum parameters were determined. Angiosarcoma tissue was investigated for chromosomal aberrations by aCGH analysis.

**Results:**

Animals of almost all different treatment regimens developed a multitude of variable liver lesions. Different tumor types such as granulation tissue type, cellular-like, hyalinization necrosis-like, angiosarcoma-like, dysplastic nodule-like, hepatocellular-like, glandular structure-like, and leiomyoma-like lesions were observed. Weekly treatment with 15 mg/kg for up to 52 weeks or a single shot of 200 mg/kg DENA led to the development of hepatic angiosarcomas. aCGH analysis of angiosarcoma tissue revealed increased alterations in tumors compared to non-tumorous tissue. Most of the chromosomal alterations were found on chromosomes 6, 7, 12, and 14.

**Conclusion:**

In this preliminary study treatment of *sus scrofa* with weekly injections of 15 mg/kg DENA results in a new model for primary hepatic angiosarcoma. This model may help to shed light on the pathomechanisms of primary hepatic angiosarcoma and might therefore open new treatment options.

## Introduction

Primary hepatic angiosarcoma is a rare malignancy, accounting for 2% of all primary hepatic tumors [1, 2]. Angiosarcoma is a highly malignant vessel tumor originating from the endothelium. Hepatic angiosarcoma is characterized by a rapid progression and poor prognosis. Treatment options are limited since angiosarcoma is resistant to chemotherapy and radiotherapy [3, 4], and is regarded as a contraindication for liver transplantation due to early recurrence and poor outcome [5].

Resection has proven to prolong survival and might therefore be curative [6, 7]. Definitive diagnosis and clinical stage are important for initial assessment [8]. However, the majority of hepatic angiosarcomas are unresectable due to late diagnosis [9]. Without treatment hepatic angiosarcoma patients anticipate a median survival of 6 months. Even after treatment, survival for more than 2 years was reported for only 3% of patients [10]. A combination of chemotherapeutic agents was suggested to improve therapeutic outcome [9].

Only few animal models are available for the study of angiosarcoma development [11, 12]. Animal models allow for the investigation of tumorigenesis and new therapeutic approaches, and can thus improve diagnosis and therapy. We presume that treatment of pigs (*Sus scrofa*) with diethylnitrosamine (DENA) can serve as a new animal model of hepatic angiosarcoma development. This model can provide new insights into human tumorigenesis of primary hepatic angiosarcoma.

## Materials and methods

### Animals

**Fig 1:**
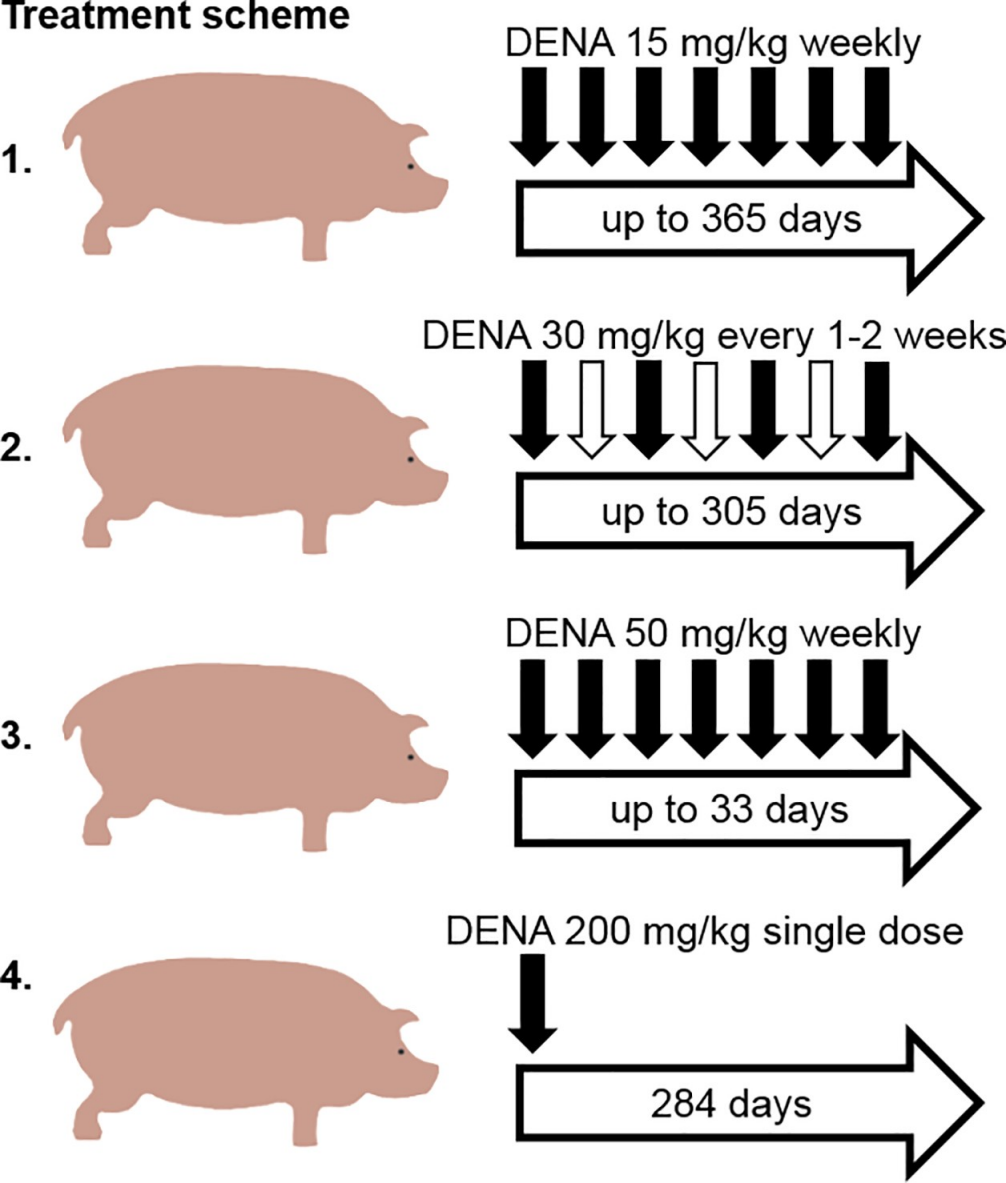
Treatment regimes. Pigs were treated with either 15 mg/kg (n = 19), 30 mg/kg (n = 2 each), 50 mg/kg (n = 4) DENA weekly or every 2 weeks. One animal was given a single dose of 200 mg/kg DENA.

All animal procedures were approved by and performed in accordance with the local animal welfare committee: Ethikkommission Medizinische Universität Graz according to the guidelines of the Austrian animal protection law. Ethical approval number is GZ66.010/006 9-II/10b/2008. Pigs *Sus scrofa* were kept in boxes of two to four animals and fed twice a day with G-22 food for fattening (Gsellmann, Austria). Diethynitrosamine (DENA, Sigma Aldrich, USA) was applied i.p. by weekly injections of 15, 30 or 50 mg DENA per kg bodyweight (Fig 1). Two animals received 30 mg DENA per kg body weight every two weeks, and one animal was injected 200 mg DENA by a single i.p. shot. Animals were sedated for the administration of DENA and for blood sampling. A short general anesthesia was maintained by application of 1 mg/kg midazolam (ERWO Pharma GmbH, Brunn am Gebirge, Austria) and 10 mg/kg ketamin (Keta-sol, aniMedica GmbH, Senden-Bösensell, Germany) intra muscular. After completing blood sampling and administration of DENA, pigs were kept in separate boxes until they woke up from narcosis. For sacrifizing animals were euthanized by an intravenous injection of 100 mg/kg pheno-barbital (Nembutal, Sanofi Ceva, Düsseldorf, Germany) by a professional veterinarian.

### Computed tomography

In order to monitor liver disease a CT scan was performed on each pig every 2–3 months. Animals were pre-medicated with 1 mg/kg midazolam (ERWO Pharma GmbH, Brunn am Gebirge, Austria), 10 mg/kg ketamin (Ketasol, aniMedica GmbH, Senden-Bösensell, Germany), and 2 mg/kg azaperon (Stresnil, Janssen-Cilag Pharma, Vienna, Austria). Animals were intubated with a 8.0 spiral tubus (Willy Rüsch GmbH, Kernen, Germany) and ventilated with an mobile ventilator (Oxylog 2000, Dräger Medical, Best, Netherlands). Sedation was maintained by a sustainable application of 5–10 mg/kg/h propofol (Diprivan, Astra Zeneca, Vienna, Austria) through a syringe pump (Perfusor F, Braun Melsungen AG, Melsungen, Germany).

CT scans were performed with a dynamic volume computed tomography scanner (Aquilion ONE, Toshiba Medical Systems, Japan). Contrast agent 2,5 ml/kg Iopamidol (Jopamiro 300mg, Bracco Austria, Vienna, Austria) was injected through the ear vein. CT images were processed with a 3D workstation (Vitrea, Toshiba Medical Systems, Japan)

### Serum parameters

Blood samples were collected every second week after first DENA injection. Blood sampling was performed either through puncture of the Port-a-Cath (PAC) if animals were equipped with PAC, or direct approach via the vena jugularis. Both methods required a short general anesthesia to guarantee safe handling. Levels of leukocytes, erythrocytes, hematocrit, hemoglobin, thrombocytes, sodium, calcium, creatine, urea, bilirubin, alkaline phosphatase (AP), gamma-glutamyltransferase (GGT), cholesterol, lactate dehydrogenase (LDH), aspartate aminotransferase (AST), alanine aminotransferase (ALT), amylase, C-reactive protein (CRP), albumin, fibrinogen, prothrombin time, and lipase were measured at the lab LB2 of the Clinical Institute of Medical and Chemical Laboratory Diagnostics, Medical University of Graz, Graz, Austria.

### Immunohistochemistry (IHC)

The primary antibodies used were specific to alpha fetoprotein (AFP; #760–2603, Roche, ready to use), p62/sesquestosome 1 (#GP62-C, Progen, dilution 1:100), Ubiquitin (#Z0458, Dako, 1:300), heat shock protein 70 (HSP70; #sc-24, Santa Cruz, 1:100), Golgi membrane protein 73 (Gp73; #sc-48011, Santa Cruz, 1:200), Ki67 (#GA626, Agilent/ Dako, ready to use), van Willebrand factor (vWF; M0616, Dako, 1:1,000), cytokeratin 5 (CK5; #AC-0181, Epitomic, 1:100), cytokeratin 8 (CK8; #CK8-TS1, Leica, 1:50), cytokeratin 18 (CK18; #MS-142-P, Thermoscientific, 1:50), alpha smooth muscle actin (SMA; #A2547, Sigma, 1: 10,000), and glutamine synthetase (GS; #MAB302, Merck/ Millipore 1:5,000). IHC was performed on a Ventana Immunostainer XT (Ventana Medical Systems, Tucson, USA) by heat-induced epitope retrieval (HIER) in cell conditioning solution for 30 min. For detection the ultra-VIEW universal DAB Detection Kit (Ventana Medical Systems, Tucson, USA) was used. Samples were examined by three independent experienced pathologists blinded to experimental conditions (J.H., C.L., and B. L.-A.).

### aCGH analysis

Paraffin-embedded liver tumors were micro-dissected and hybridized against age-matched control liver tissues. Labeling was performed according to the BioPrime aCGH Genomic Labeling Module protocol (Invitrogen). The samples were hybridized on an 8x60k CGH Array under the conditions of the Agilent protocol (Version 7.2). The arrays were analyzed with an Agilent DNA Microarray Scanner G2505C and the extraction software Agilent Feature Extraction 11.0.1.1.

### Statistics

Data analysis and statistics of experimental data were performed using Origin software (OriginPro 8.1G; OriginLabs). Statistical differences in serum parameters were estimated by one-sample t-test against 0% change after verification of normal distribution by the Shapiro-Wilk method. Correlation analysis was performed using Pearson correlation. All tests were considered statistically significant when *p* values were less than 0.05.

## Results

**Fig 2:**
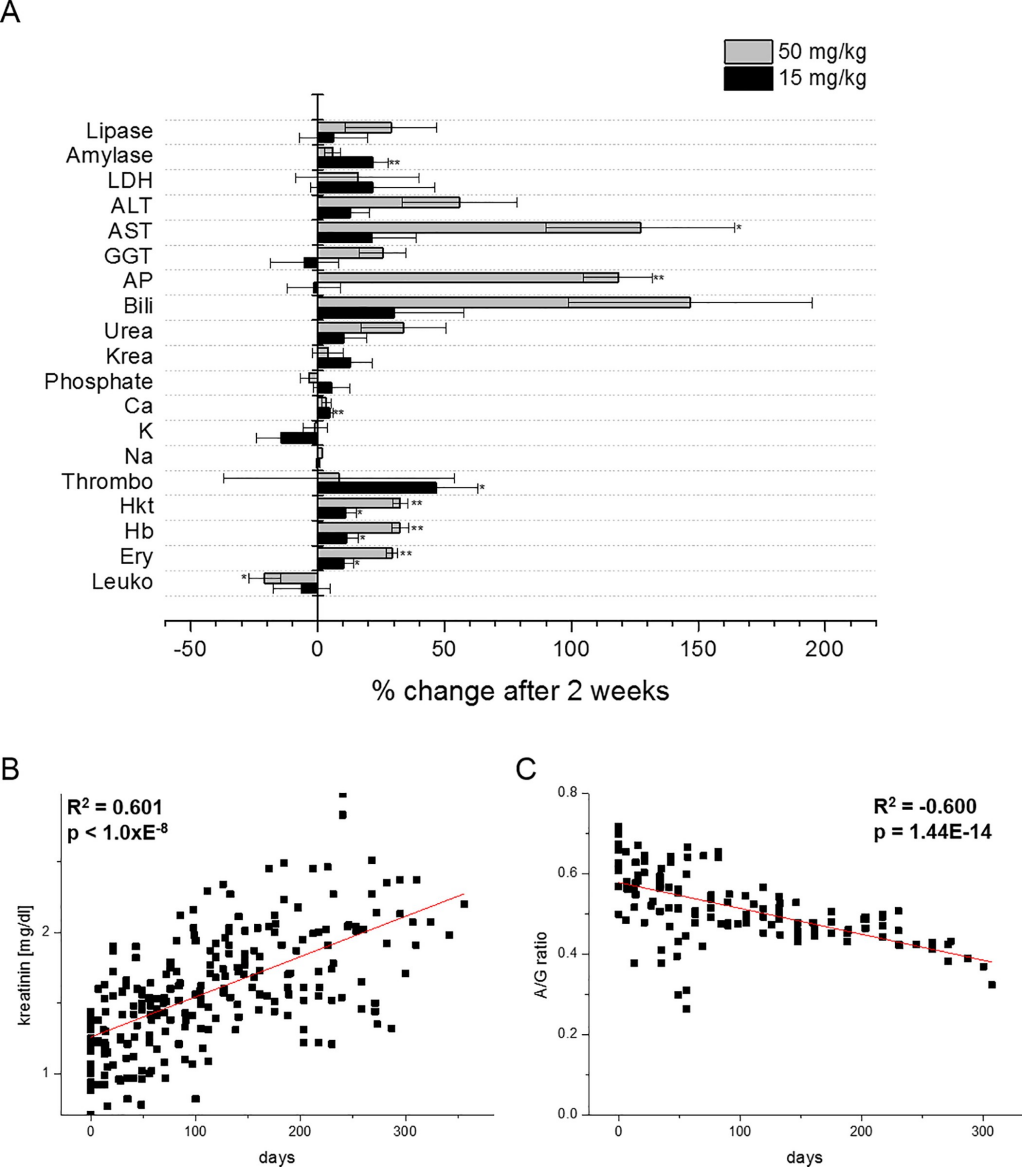
Serum parameters of pigs treated with either 15 mg/kg or 50 mg/kg. A) Relative changes of serum parameters are shown in pigs treated with 15 mg/kg or 50 mg/kg two weeks after first DENA administration. Data were normalized to day zero and are shown as percentage. B,C) Scatterplots of kreatinin (B) and albumin/total serum protein ratio (A/T) (C) against treatment time are shown for pigs treated with 15 mg/kg DENA. Pearson correlation coefficients are given.

Different treatment regimens were used for hepatic tumor induction (Fig 1). Serum analysis revealed altered amylase, calcium, hematocrit, hemoglobin, and erythrocytes in the 50 mg regimen two weeks after first DENA injection (Fig 2A). In the 15 mg regimen, AST, AP, hematocrit, hemoglobin, and erythrocyte levels were increased (Fig 2A). Over the whole duration of treatment, kreatinin levels significantly increased with time (Fig 2B), and the albumin to total serum protein ratio (A/T) decreased (Fig 2C).

**Fig 3:**
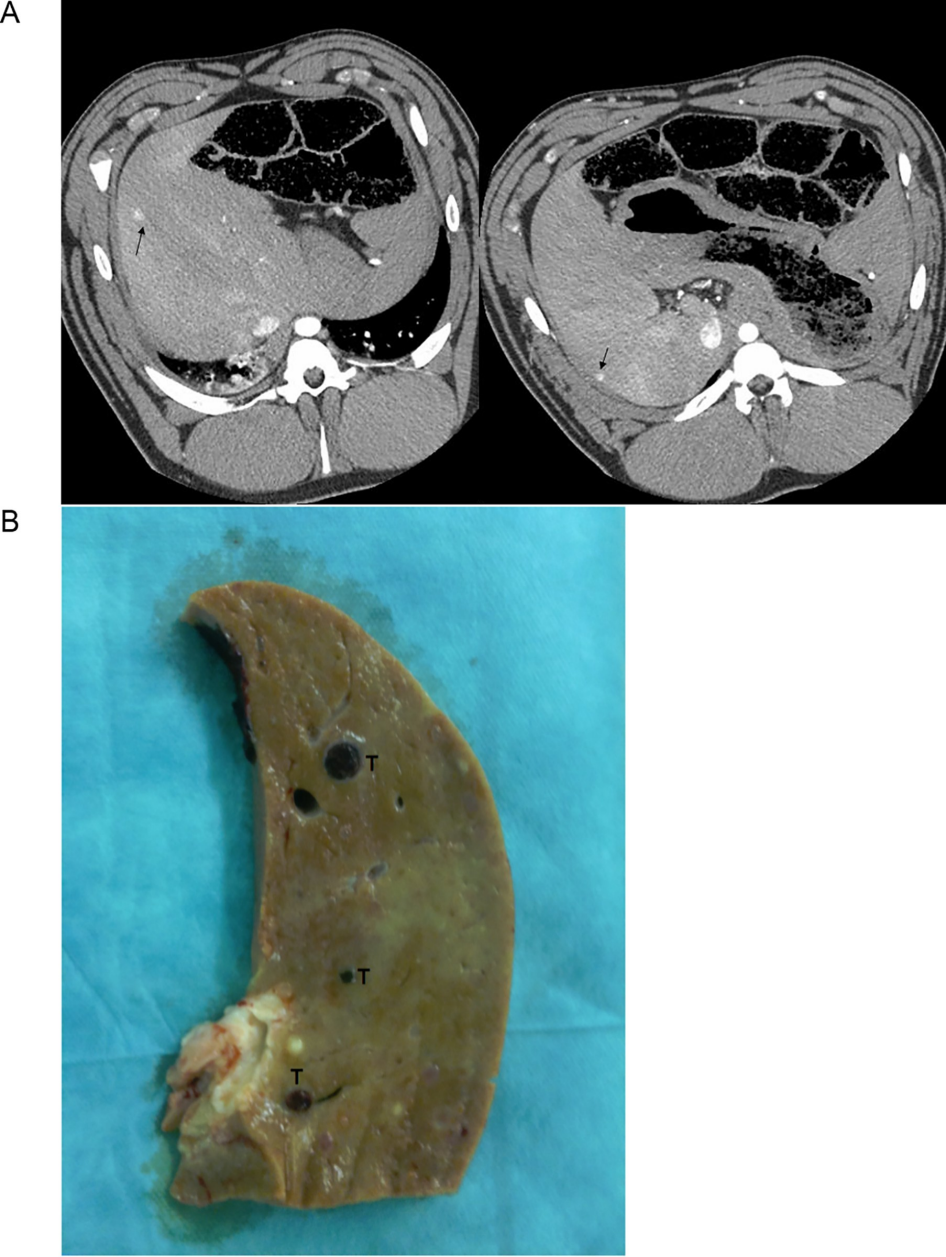
CT-Scans and macroscopic visualization of liver lesions. A) CT scans performed according to the protocols for a pig treated with 15 mg/kg DENA for 90 days. Arrows indicate radiologically verified liver tumor in the pig liver. B) Macroscopic picture from a transverse liver section of a pig treated with 15 mg/kg DENA. Tumor lesions (T) of different colors and sizes are indicated.

**Fig 4:**
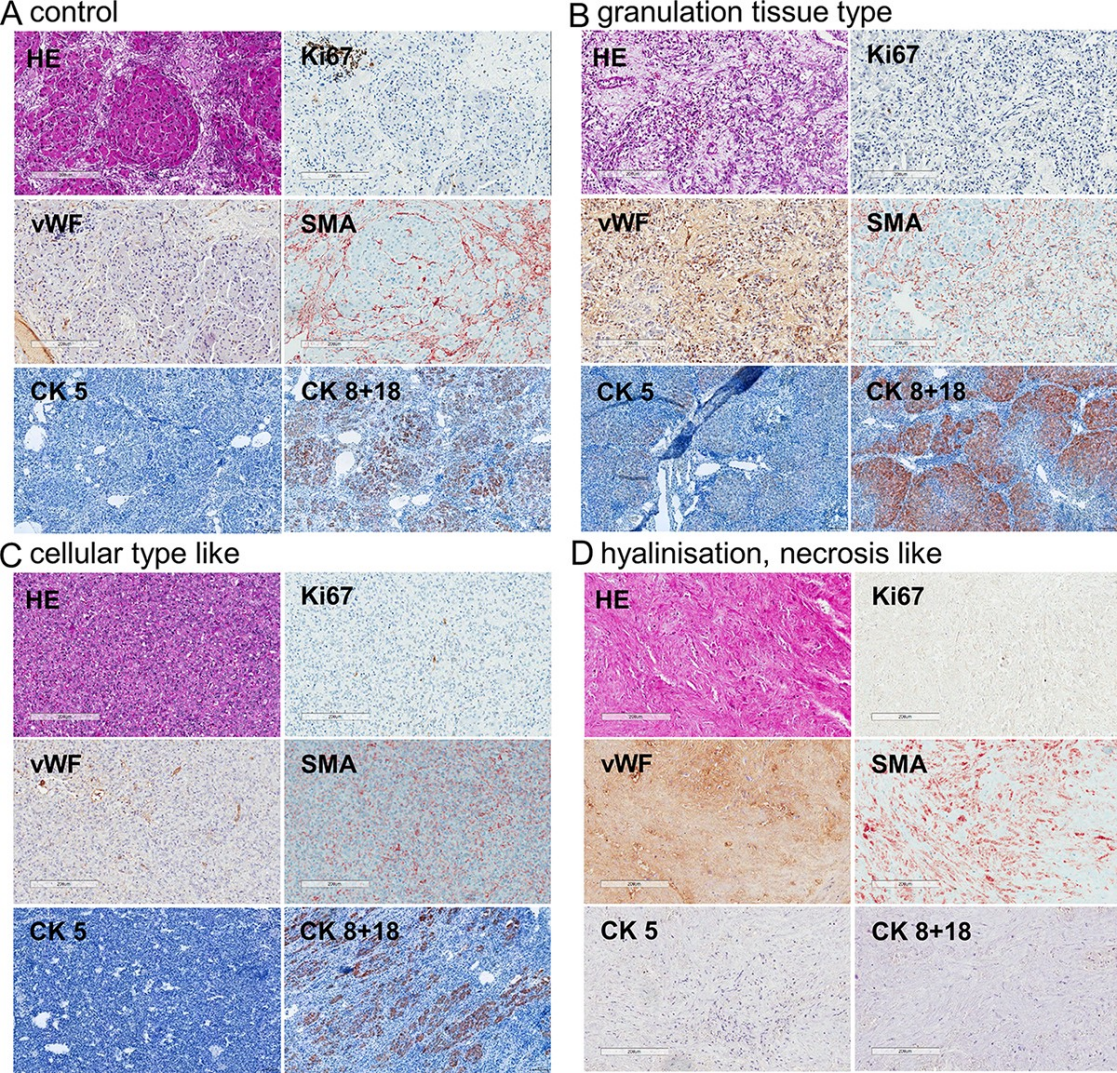
Histology of the different liver lesions. Representative images of treatment group 1 (15 mg/kg DENA weekly) are shown. A) Control, B) granulation tissue type, C) cellular type like, and D) hyalinization like. Ki67, van Willebrand factor (vWF), cytokeratin 8 and 18 (CK8+18), CK5, and alpha smooth muscle actin (SMA). Scale bars: 200 μm or 100 μm for CK5 and CK8+18 in A, B, and C.

**Fig 5:**
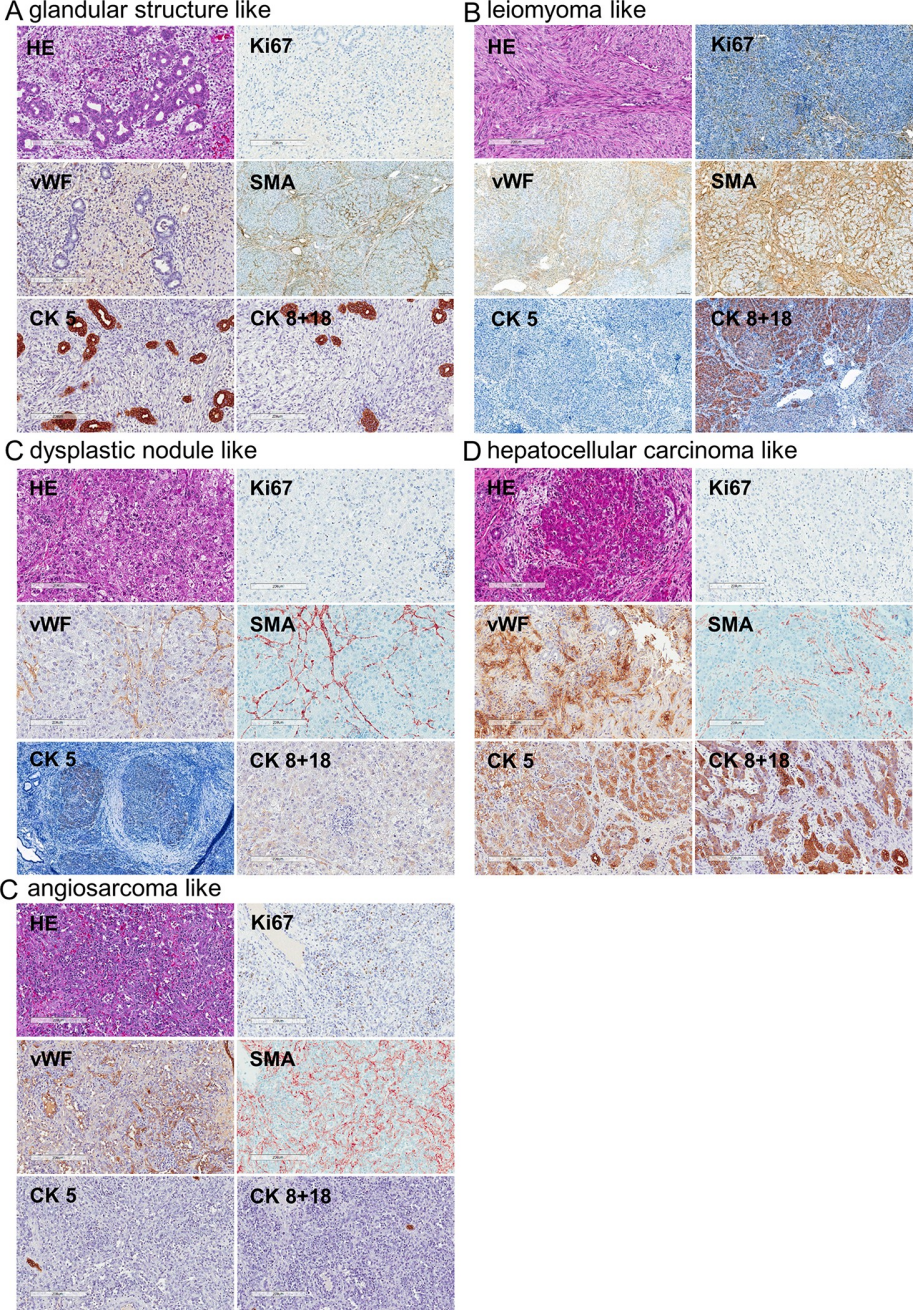
Histology of the different liver lesions. Representative images of treatment group 1 (15 mg/kg DENA weekly) are shown. A) Glandular structure like, B) leiomyoma like tumors C) dysplastic nodule like, D) hepatocellular carcinoma like, and E) angiosarcoma like tumors. Ki67, van Willebrand factor (vWF), cytokeratin 8 and 18 (CK8+18), CK5, and alpha smooth muscle actin (SMA). Scale bars: 200 μm or 100 μm for SMA in A, Ki67, vWF, SMA, CK5, and CK8+18 in B, and CK5 in C.

Development of cirrhosis was observed in 42% of the animals independent of the respective treatment regimen. All of the long-term DENA-treated animals developed liver lesions (Fig 3). Only the animals treated with 50 mg/kg did not develop any liver lesions, which is probably due to the short treatment duration of up to four weeks. Histological evaluation of the liver lesions revealed the following different tumor types: granulation tissue type (Fig 4B), cellular-like (Fig 4C), hyalinization necrosis like (Fig 4D), lymphoma-like with glandular structure (Fig 5A), and leiomyoma-like (Fig 5B), dysplastic nodule-like (Fig 5C), hepatocellular-like (Fig 5D), and well defined mesenchymal lesions with morphologic features reminiscent of angiosarcoma (Fig 5E). The most common liver lesions were dysplastic nodules (21.9%), angiosarcoma-like tumors (18.8%), and HCC-like tumors (21.9%) (lymphoma-like with glandular structure: 12.5%; granulation tissue type, cellular-like, hyalinization necrosis like: 6.3% each; leiomyoma-like and tumors with spindle cells and hepatocyte-like cells: 3.1% each). The angiosarcoma-like tumor type developed in animals treated with either 15 mg/kg DENA weekly or in the animal subjected to a single i.p. injection of 200 mg/kg. In pigs treated with the latter regime, also granulation tissue type, hyalinization-like, and dysplastic nodule-like lesions were observed. Animals treated with 30 mg/kg DENA developed cellular-like and hyalinization-like lesions only.

**Fig 6:**
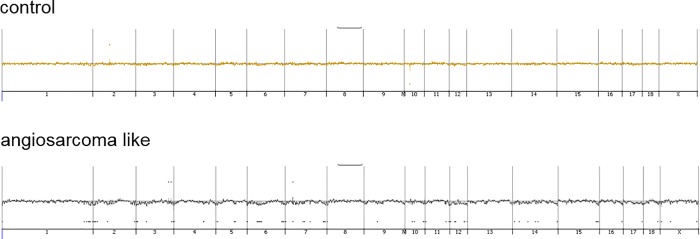
aCGH analysis of pig angiosarcoma. Representative aCGH plots of hepatic angiosarcoma (lower panel) and control tissue (upper panel). Chromosomal aberrations are shown along the chromosomes.

In order to study genetic events in pig angiosarcoma, an aCGH analysis was performed. Increased alterations in tumors, compared to non-tumorous tissue, were observed (Fig 6). The majority of these alterations were found on chromosomes 6, 7, 12, and 14. Interestingly, porcine chromosome 12 corresponds to human chromosome 14 and carries the tumor suppressor gene *TP53*. Moreover, the tumor suppressor *PTEN* is located on porcine chromosome 14 or on the human homologue chromosome 10.

## Discussion

Primary hepatic angiosarcoma is a rare, but rapidly progressing tumor with poor prognosis. There is no established chemotherapy scheme, and hepatic angiosarcoma is *per se* resistant to chemotherapy and radiotherapy.[3, 4] Targeted therapy might lead to advances in angiosarcoma therapy, but preclinical and clinical studies are still ongoing [13].

In this study, we describe a novel and easy model to induce hepatic tumor lesions in pigs modeling human primary hepatic angiosarcoma by weekly i.p. injections of 15 mg DENA per kg body weight. Immunohistochemical analysis revealed positivity against vWF as an endothelial cell marker. Staining against endothelial markers, such as vWF, CD31, or CD34, is commonly used for the diagnosis of human hepatic angiosarcoma [14]. Also, SMA positivity has been described in case-reports to be a marker for angiosarcoma [15] and Ki67 proliferative index has been reported to range between 10 and 60% [14].

Animal models for hepatic angiosarcoma are rare–only few models are available [11, 12]. The most recent progress in angiosarcoma models was presented by Rothweiler et al.: The authors established an angiosarcoma cell line which can be used for a xenograft model, which allows an easy and fast screening of potential therapeutics *in vivo* [16]. Although xenograft models display a convenient tool to study tumor progression, they do not allow for the study of mechanisms triggering early processes in carcinogenesis.

To the best of our knowledge, this is the first study investigating chromosomal aberrations in primary hepatic angiosarcoma. Verbeke and colleagues conducted a CGH analysis on thirteen human angiosarcomas of bone and five human angiosarcomas of soft tissue [17]. The latter study presented two subgroups of human angiosarcoma, i.e. one with either no (5/18 samples) or few or weak (9/18 samples) aberrations and one with numerous genetic aberrations (4/18 samples) [17]. Interestingly, five of the human angiosarcomas investigated by Verbeke et al. displayed almost exclusively deletions and no or few amplifications,[17] which is in accordance with our study of porcine hepatic angiosarcoma. Mutations in the *TP53* gene have been reported in a small cohort of patients affected by human hepatic angiosarcoma [18]. Furthermore, mutations of the tumor suppressor *PTEN* have been reported to occur in human hepatic angiosarcoma [19]. In contrast, however, a study investigating 30 primary and 32 secondary human cases of angiosarcoma detected no deletions in the *TP53* and the *PTEN* genes [20]. However, the cohort of the latter study contained only one sample of hepatic angiosarcoma, suggesting different chromosomal aberration profiles in angiosarcomas of different origin. Due to the heterogeneity of human angiosarcomas that are dependent on their primary site of disease [21], results of studies investigating cohorts, including angiosarcomas from different tissues, must not be applied to primary hepatic angiosarcoma. Still, the authors observed a frequent activation of the PIK3/AKT/mTOR pathway [20]. Thus, genetically engineered models recapitulating *TP53* and *PTEN* deletions, as well as PI3K/AKT/mTOR activation, might be helpful as additional tools for the study of angiosarcoma development [22], although chemically induced models rather reflect the origin of carcinogenesis in humans. Further studies investigating chromosomal aberrations in human hepatic angiosarcoma are needed to compare the alterations seen in the DENA model with human malignancy.

In conclusion we present an easy model to induce hepatic angiosarcoma in pigs and give a first insight in chromosomal aberrations of these lesions. Future studies will reveal a more detailed picture of the molecular events in this model.

## Abbreviations

DENA: diethylnitrosamine
CT: computed tomography
aCGH: array-based comparative genomic hybridization
PAC: Port-a-Cath
AP: alkaline phosphatase
GGT: gamma-glutamyltransferase
LDH: lactate dehydrogenase
AST: aspartate aminotransferase
ALT: alanine aminotransferase
CRP: amylase, C-reactive protein
AFP: alpha fetoprotein
HSP70: heat shock protein 70
Gp73: Golgi membrane protein 73
vWF: van Willebrand factor
CK5+8: cytokeratin 5 and 8
SMA: alpha smooth muscle actin
GS: glutamine synthetase
AEC: amino ethyl carbazol
DAB: 3,3'-diaminobenzidine
A/T: albumin to total serum protein ratio
